# Supplementary material for: Brain-derived neurotrophic factor derived from sensory neurons plays a critical role in chronic pain
Source: Brain. 2018 Jan 30;141(4):1028–39. doi: 10.1093/brain/awy009 (PMC5888992; doi:10.1093/brain/awy009)
Supplement: Supplementary Figure S1 [file brain-2017-01051-file008_awy009.pdf]

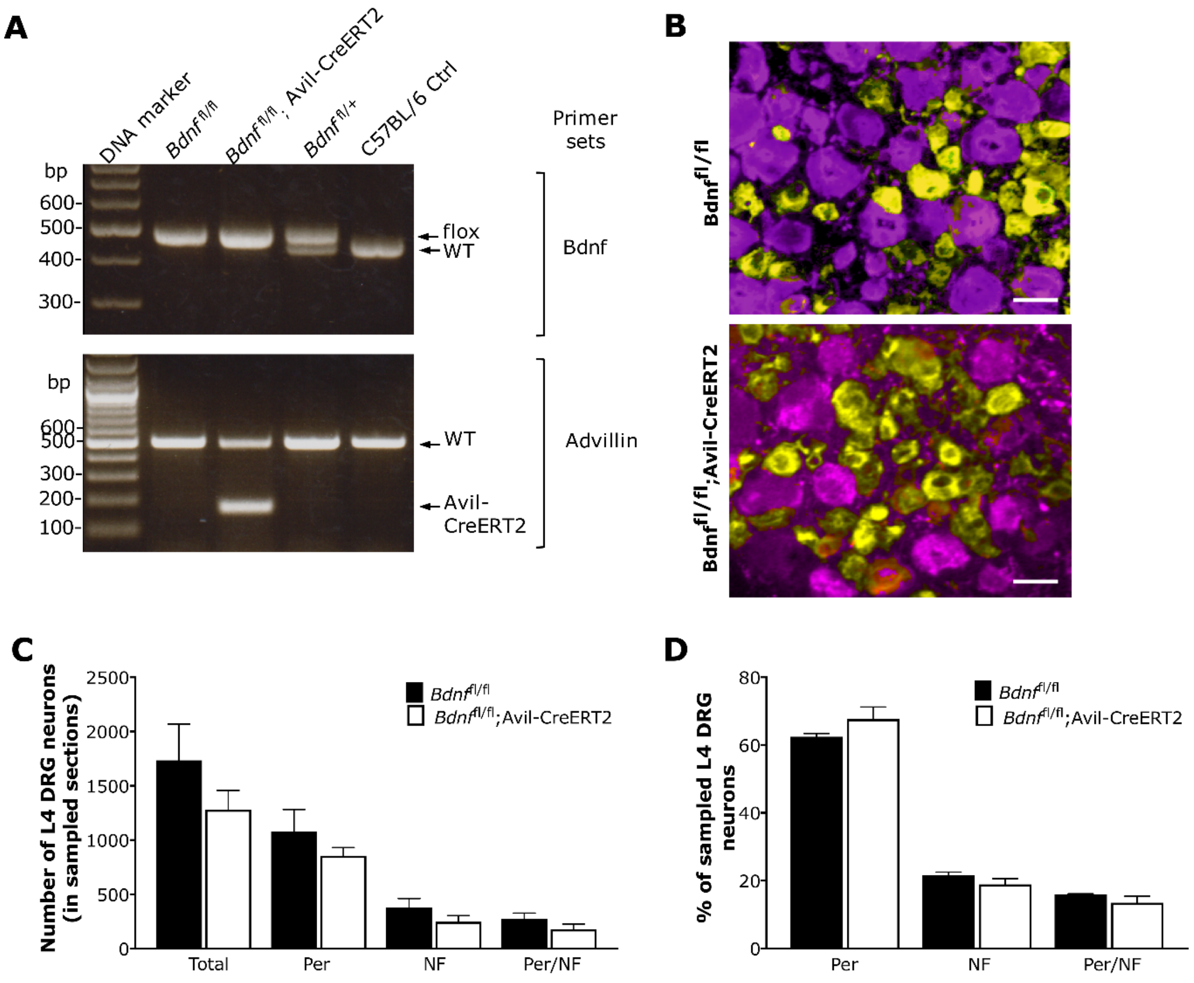


## Supplemental Figure S1.

**Characterization of BDNF knockout mice.** (**A**) Genotyping analysis with PCR. The representative gel analysis of PCR products using both the BDNF primer set and the Advillin-CreERT2 primer set is shown in top and bottom panels, respectively. Mice homozygous for the floxed *Bdnf* band and heterozygous for the Advillin-CreERT2 band were defined as *Bdnf*$^{fl/fl}$; Avil-CreERT2 mice. Mice only homozygous for the floxed Bdnf band were defined as *Bdnf*$^{fl/fl}$ littermate controls. The genomic DNA from C57BL/6J wild-type mice and heterozygous floxed Bdnf (*Bdnf*$^{fl/+}$) mice were used as controls. (**B**) DRG sections were labelled with large diameter DRG neuron marker neurofilament (in magenta), and small-medium diameter DRG neuron marker peripherin (in yellow). Scale bar, 50 µm. (**C**) The number of DRG neurons either expressing neurofilament (NF), or peripherin (Per) in the sampled L4 DRG sections, or both NF and Per were counted respectively. (**D**) The proportions of neurofilament and peripherin positive neurons in L4 DRG are presented. Both total number and proportion are normal in BDNF knockout mice ($n$ = 3) compared to littermate controls ($n$ = 3). Data were analysed with Student's $t$-test and $P > 0.05$.
